# Central Hypercapnia in a Neonate With Parechovirus Infection

**DOI:** 10.7759/cureus.67455

**Published:** 2024-08-22

**Authors:** Ryo Tahata, Akio Yamano, Masashi Zuiki, Yasunori Ishihara, Shinji Akioka

**Affiliations:** 1 Pediatrics, Fukui Aiiku Hospital, Fukui, JPN; 2 Pediatrics, Kyoto Prefectural University of Medicine, Kyoto, JPN

**Keywords:** high-flow nasal cannula (hfnc), neonates, hypercapnia, human parechovirus, central neurological dysfunction, ventilation failure

## Abstract

Human parechovirus infections in newborns often affect the central nervous system. It is common in children after infancy for it to be a cause of the common cold or be asymptomatic, but an infection in infancy often causes a central nervous system infection. Herein, we present the case of a nine-day-old infant who developed hypercapnia without any involvement of respiratory lesions. She showed no hypoxia or circulatory abnormalities. A high-flow nasal cannula relieved hypercapnia and consequent respiratory acidosis, suggesting that the hypercapnia was due to central ventilation failure with central nervous system infection despite no abnormalities on brain magnetic resonance imaging. Accurate diagnosis and intervention of ventilatory failure, which is a central nervous system dysfunction, is important in hypercapnia associated with parechovirus infection.

## Introduction

Human parechovirus (HPeV) infections occur predominantly in early childhood and present with various clinical manifestations [1]. Most cases are subclinical and have good prognoses [2]; however, infections during the neonatal period or early infancy can lead to severe organ damage such as encephalitis, meningitis, and sepsis-like syndrome [3,4]. Moreover, encephalitis can lead to neurological sequelae and fatal outcomes [5,6].

Herein, we report a case of HPeV infection in a neonate who presented with hypercapnia and tachypnea. Hypercapnia, a rare manifestation of HPeV infection, is caused by a central ventilation defect without airway involvement [7]. In this case, a high-flow nasal cannula (HFNC) with ambient air relieved the ventilatory disturbance. This case report suggests that disturbances in the central nervous system are variably manifested in neonatal HPeV infection such as central respiratory failure. Adequate understanding of the pathogenesis and appropriate treatment are essential to prevent organ damage in such cases.

## Case presentation

A nine-day-old girl was referred to our hospital with a fever. She fed poorly the night before and developed a fever on the day of admission. She was born following 39 weeks and 6 days of gestation via vaginal delivery. Her birth weight was 3024 g and her Apgar scores were 8 at 1 minute and 9 at 5 minutes. The mother had no apparent infection or abnormal course during the pregnancy and delivery. She was discharged from the maternity hospital when she was five days old.

On admission, the patient weighed 3090 g; her temperature was 38.7°C, her pulse rate was 168 beats/min, systolic and diastolic blood pressures were 78 and 43 mmHg, respectively, and her respiratory rate was 50 breaths/min. Although her respiration was shallow, retractive, or forced breathing, as well as abnormal breaths, such as wheezing, were not observed. Transcutaneous oxygen saturation was 95% in ambient air. Although appearing to be not doing well, she was capable of crying as usual and had no body movement impairments. Her peripheral skin was cold but not cyanotic, anemic, or mottled. Auscultation revealed no abnormal cardiac or respiratory sounds. Her abdomen was slightly distended; however, intestinal peristalsis was audible.

**Figure 1:**
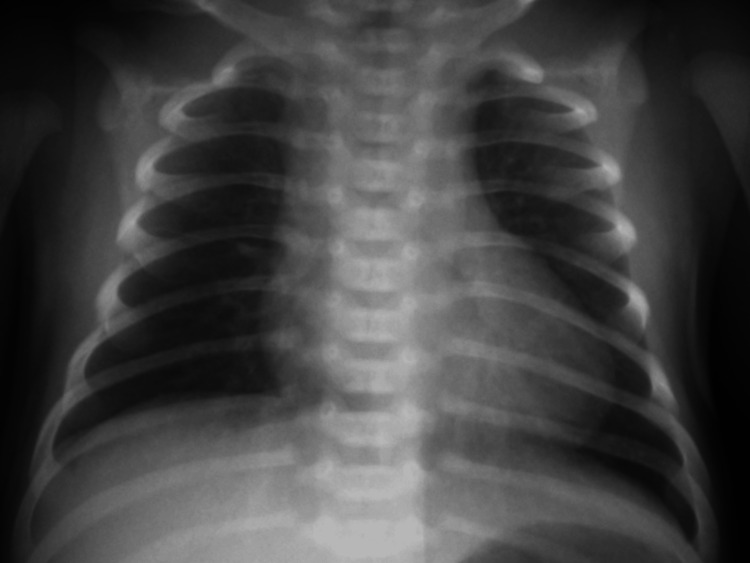
Chest radiography Chest radiography showed no abnormalities.

Venous blood gas analysis revealed respiratory acidosis (pH 7.275), mildly elevated partial pressure of CO_2_ (pCO_2_) of 54.0 mmHg, bicarbonate (HCO_3_^-^) of 21.5 mmHg, BE of -2.3 mmol/L and lactate of 2.92 mmol/L. Her blood glucose level was 96 mg/dL. Her white blood cell count was 7500/microL, neutrophil count was 4280/microL, and C-reactive protein level was 0.3 mg/dL; no abnormalities were noted on blood biochemistry and urinalysis. A cerebrospinal fluid test showed a cell count of 3/mm^3^, protein level of 50 mg/dL, and sugar level of 64 mg/dL. Bacterial cultures of blood, urine, spinal fluid, and nasal cavity were negative. Antigen tests for respiratory syncytial virus, SARS-CoV-2, and influenza viruses in the nasal fluid were negative. Chest radiography revealed no abnormalities (Figure 1).

**Figure 2:**
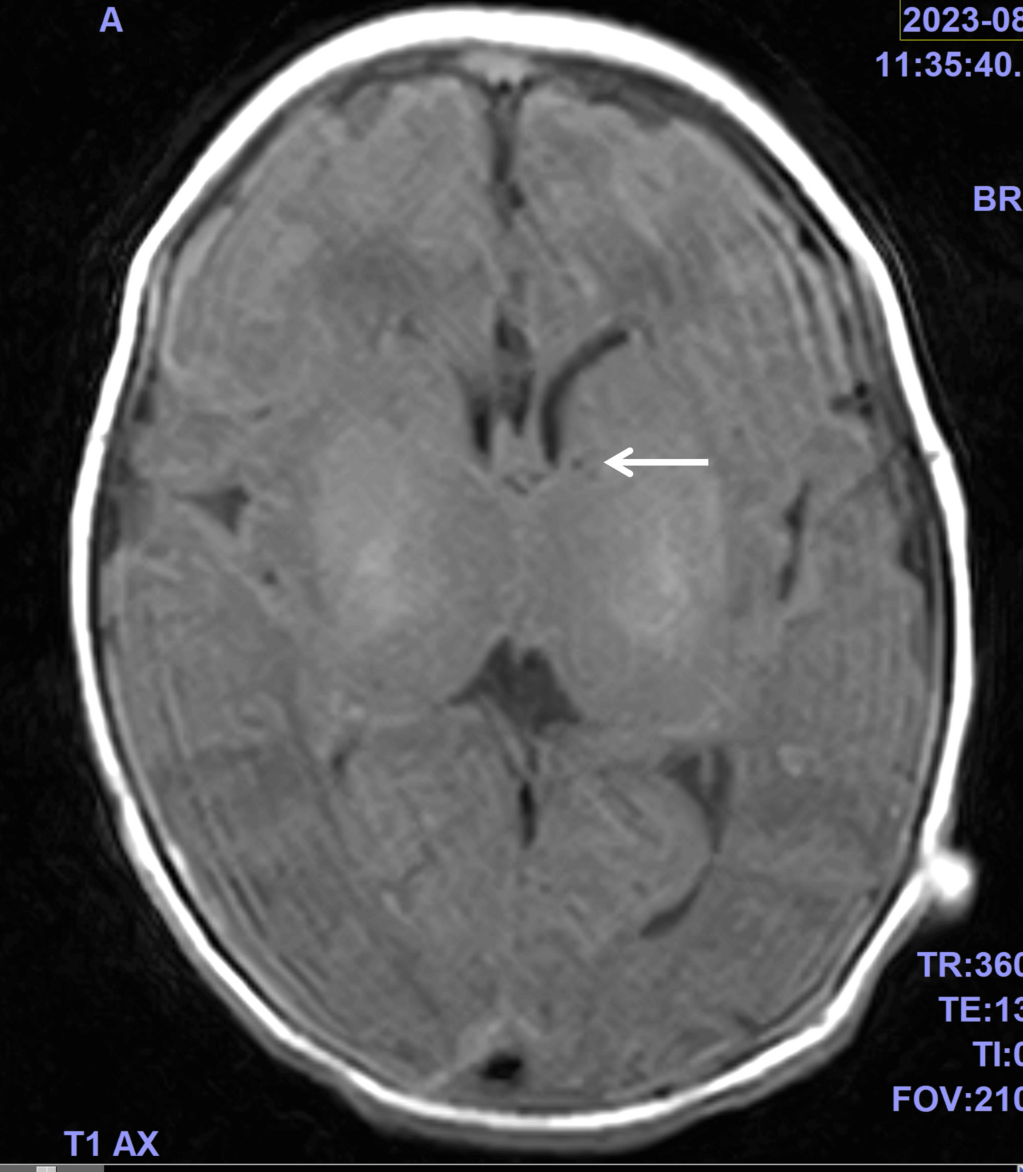
Axial T1-weighted magnetic resonance imaging of the brain Axial T1-weighted magnetic resonance imaging of the brain showed no abnormal findings except for subependymal cysts (indicated by a white arrow).

The patient was admitted to the hospital with a fever. At this point, the cause of the fever and tachypnea was not apparent. Three hours after admission, her respiratory rate decreased to 34/min but pCO_2_ increased to 87.4 mmHg with a pH of 7.051, indicating worsening respiratory acidosis. After an additional 4 h, pCO_2_ and pH recovered to normal levels without interventions; however, after another 15 h, CO_2_ retention, and respiratory acidosis reappeared without a marked increase in the respiratory rate. Uncontrolled respiration, persistent tachycardia, and severe coldness were deemed to indicate impairments of the central nervous system (CNS), including the respiratory center. Results of repeat cerebrospinal fluid tests were unremarkable; however, the HPeV genome was detected in a microarray assay. Brain magnetic resonance imaging (MRI) showed no abnormal findings except for subependymal cysts, which appeared to be congenital, as no changes were observed in follow-up MRIs (Figure 2).

She was diagnosed with HPeV infection with a central ventilation defect. HFNC with ambient air was initiated based on the hypothesis that hypercapnia was due to reduced minute ventilation related to central ventilation defects. Two hours later, HFNC successfully reduced pCO_2_ to 54.5 mmHg and after another 2 hours, normalized it to 38.7 mmHg (Table 1).

**Table 1:** Blood gas values and vital parameters

Parameters	Time after admission, hours							
	0	3	7	22	29	49	69	144
Respiratory support	No	No	No	No	HFNC	HFNC	HFNC	No
Blood gases								
pH	7.275	7.051	7.385	7.126	7.386	7.379	7.413	7.379
pCO_2_, mmHg	54.0	87.4	38.1	73.3	38.7	43.6	43.5	38.5
HCO_3_^-^, mmol/L	21.5	16.4	22.4	18.0	22.6	24.1	26.2	22.0
Vital parameters								
SpO_2_, %	95	98	98	100	100	97	96	98
Respiratory rate, min^-1^	50	34	45	38	28	30	38	47
Heart rate, min^-1^	168	174	175	162	153	144	156	146
Temperature, ℃	38.7	38.2	37.7	38.1	37.6	37.1	36.5	36.5

Hypercapnia disappeared after the introduction of HFNC. The next day, her respiratory rate decreased to 30 breaths/min, the fever disappeared, and her general condition improved. Tachycardia resolved gradually and diminished on day 6 post-admission; subsequently, HFNC was discontinued.

Repeat MRI examinations one and three months later revealed no abnormalities in the brain. Currently, the patient is seven months old and without apparent motor or mental disabilities.

## Discussion

Various microorganisms, including group B *streptococcus* and *Escherichia coli*, can be involved in fever in the neonatal period and early infancy. A notable infectious disease is the HPeV infection. In a prospective study, HPeV was identified in 19.6% of cases of fever of less than 3 months in which a pathogenic virus was identified [8]. The study also noted that HPeV tends to be detected from July to September, which applied to the present case.

HPeV infections exhibit diverse clinical presentations. Infections in neonates are symptomatic and present with fever, moodiness, feeding difficulties, tachycardia, abdominal distension, and erythematous palms [1]. Severe cases, such as septic shock syndrome or encephalitis, are particularly noteworthy. However, as a newly recognized infectious disease, epidemiological studies are in progress; hence, the risk factors for severe phenotypes of HPeV infections remain unclear. The present case had a typical clinical presentation of neonatal HPeV infection, except for hypercapnia without dyspnea, which to our knowledge, has not been reported previously.

HPeV rarely causes lower respiratory tract infections; however, the viral genome has been detected in the airways. Respiratory failure is a major cause of admission to the pediatric intensive care unit [9]. In a 10-year prospective observational study, respiratory failure was observed in two-thirds of patients with encephalitis [4]. Another study reported that more than half of patients with encephalitis had respiratory symptoms [10]. These findings suggest that respiratory symptoms may be a sign of severe HPeV infection and a reflection of extra-respiratory organ failure.

In this case, hypercapnia without hypoxia was observed. Direct HPeV involvement in respiratory areas, such as pneumonitis, was not evident on imaging, indicating that hypercapnia was caused by a reduction in ventilation volume. Decreased ventilation volume can be caused by a reduction in minute ventilation or frequency of ventilation. As the patient presented with tachypnea, decreased minute ventilation was considered the main cause of hypercapnia. Minute ventilation is influenced by respiratory muscle movement. As hypotonia is a symptom of HPeV infection [4], impaired movement of the respiratory muscles may cause hypercapnia. The respiratory center in the brainstem physiologically monitors blood pCO_2_ levels via chemoreceptors and is involved in respiratory control. Herein, the lack of respiratory upregulation under hypercapnia may have been a central ventilation defect. Although neonates with respiratory syncytial virus infection are prone to central apnea [11], the pathophysiologic mechanisms responsible for this symptom remain unclear. Some previous studies have shown that a prolonged laryngeal chemoreflex induced by disruptions in the neural control pathways, or an involvement of inflammation in the CNS, are the causes of apnea in neonates infected with this virus. As apnea attacks have already been reported in HPeV infections [12], a similar defect in respiratory control was considered in the present case.

HPeV has a high affinity for the CNS, and infections often result in encephalitis. In Japanese infants, encephalitis has been reported in >10% of infected cases [13]. The manifestations include convulsions, poor overall status, altered mental status, and signs of circulatory failure. MRI studies have shown diffusion abnormalities in the periventricular and subcortical white matter, corpus callosum, internal and external capsules, and pyramidal tracts [4]. Herein, no CNS involvement, such as encephalitis, was evident as per the manifestations and in imaging studies using MRI. However, based on the presence of a central ventilation defect, this case was deemed equivalent to encephalitis. Various electroencephalogram abnormalities, including subclinical seizures, are frequently observed in HPeV infection [14], regardless of whether abnormalities are observed in MRI findings [13]. In the present case, the transient central ventilation defect may have been caused by intermittent subclinical seizures at the respiratory center, resulting in recurrent hypercapnia.

The prognosis of parechovirus infection remains unclear, and there is no curative treatment for HPeV encephalitis; only symptomatic treatment is available for organ protection. In cases of convulsions and hypotension, anticonvulsants and pressure-raising drugs are administered, respectively. For enterovirus, which is also a picornavirus infection, the antiviral drug Pleconaril has been developed. Currently, there are no antiviral drugs for parechovirus; therefore, it is expected that antiviral drugs against parechovirus will be developed.

In the present case, hypercapnia and respiratory acidosis due to central ventilation defects were treated using forced inspiration with HFNC [15]. Ventilation is regulated at the respiratory center via signals provided by CO_2_-sensitive chemoreceptors or the vagal reflex from alveolar stretch receptors. As the regulation via CO_2_-sensitive chemoreceptors was deemed impaired herein, the Hering-Breuer reflex using the latter system was utilized; the Hering-Breuer reflex is initiated by lung expansion-excited stretch receptors [16]. Stimulation of the respiratory center via the vagal nerve shortens the inspiratory time and accelerates breathing frequency. Forced inspiration with HFNC successfully resolved central hypoventilation with hypercapnia. This confirms the usefulness of respiratory therapy via the physiological vagal reflex rather than forced mechanical ventilation.

## Conclusions

Herein, our patient was diagnosed with HPeV infection based on typical presentation and a microarray analysis. Additionally, the patient exhibited hypercapnia without dyspnea, which, to our knowledge, is a novel presentation of the disease. The patient was successfully treated using HFNC.

In HPeV infections in neonates, impaired ventilation can be due to a CNS dysfunction that should not be overlooked. Based on the pathophysiological condition, appropriate therapeutic intervention is important to relieve the dysfunction and achieve neurological integrity.
